# Special Issue “Infective Endocarditis: What Is New in the Clinical Research?”

**DOI:** 10.3390/jcm12155064

**Published:** 2023-08-01

**Authors:** Petros Ioannou

**Affiliations:** 1 School of Medicine, University of Crete, 71003 Heraklion, Greece; p.ioannou@uoc.gr; 2 Internal Medicine Department, University Hospital of Heraklion, 71110 Heraklion, Greece

Infective Endocarditis (IE) is a disease that carries high morbidity and mortality risks and involves the infection of the endocardium, and more commonly of the cardiac valves and prosthetic material, like implantable defibrillators or pacemakers [1,2]. The epidemiological characteristics and the microbiology of this disease have changed considerably during the last decades [3,4]. This has led to a significant revision of the diagnostic criteria, which has led to increased sensitivity for the diagnosis of IE to allow the implementation of the changing profile of the disease, as well as the implementation of newer methods such as positron emission tomography or next-generation metagenomic sequencing [5,6,7]. Thus, ongoing clinical research on IE is critical to allow the timely identification of changes in epidemiology and microbiology. This Special Issue of the *Journal of Clinical Medicine* (*JCM*) aimed to collect high-quality scientific contributions on the topic of clinical research in IE.

In their original study, Asai et al. tried to clarify whether there is a difference among the profiles and clinical outcomes of patients who are eligible and those who are not eligible for enrollment in a randomized controlled trial (RCT) [8]. All patients in their hospital from 2007 to 2019 with a diagnosis of IE were evaluated. Patients were divided into two groups, those who were eligible for enrollment in an RCT (the RCT-appropriate group) and those who were not (the RCT-inappropriate group), based on exclusion criteria determined from previously published clinical trials. In total, 66 patients with a median age of 70 years were enrolled in the study, and 70% were male. The RCT-inappropriate group consisted of 74% of the patients. Patients in the RCT-inappropriate group were older and had more comorbidities. Compared with the patients in the RCT-inappropriate group, those in the RCT-appropriate group had a milder form of the disease and also had significantly longer overall survival. Thus, this study suggests that there is a significant gap in the characteristics of patients and clinical outcomes between patients in RCTs and those who are not eligible for inclusion in RCT, implying that physicians should be aware that RCTs may not be completely representative of the real-world population. 

Calderon-Parra et al. aimed to assess the risk of IE after surgical aortic valve replacement (SAVR) compared with transcatheter aortic valve implantation (TAVI) [9]. They prospectively followed a cohort of patients with TAVI/SAVR from March 2015 to December 2020, and performed a propensity-score-based analysis to include variables previously associated with TAVI/SAVR and IE. In total, 355 patients with SAVR and 278 patients with TAVI were included in the analysis. The median follow-up was similar between the two patient groups. IE occurred more frequently in patients with TAVI (4.65%) compared to patients with SAVR (1.41%). TAVI patients were more likely to have early IE compared to patients with SAVR. In the propensity score analysis, the risk for IE was similar. Younger age, complicated diabetes mellitus, chronic obstructive pulmonary disease, advanced heart failure, and peripheral arteriopathy were factors associated with TAVI IE.

*Mycoplasma hominis* is a microorganism that may colonize the lower genital tract and may cause genital or neonatal infections [10]. Bustos-Merlo et al. present a case of *M. hominis* IE and review the literature about this rare clinical entity [11]. More specifically, a 67-year-old male farmer with a history of arterial hypertension, hypercholesterolemia, and asymptomatic hyperuricemia developed sustained monomorphic ventricular tachycardia secondary to structural heart disease and was treated with ablation and the placement of an implantable cardioverter-defibrillator (ICD). He developed signs of sepsis in the context of an infection of the ICD generator pocket a few days later, and was admitted to the hospital. He then underwent cardiovascular surgery for the explantation of the ICD, pericardiocentesis, in which 900 cc of exudate fluid was aspirated, and debridement of the generator pocket. The fluid was positive for *Mycoplasma* spp. and SeptiFast (LightCycler^®^ SeptiFast Test Mgrade; Roche Diagnostics GmbH, Mannheim, Germany) was positive for *M. hominis* in a blood culture. Moreover, the pericardial fluid, the pocket wound exudate and the ICD lead were all positive for *M. hominis*. The patient was treated with antimicrobials, a new ICD was placed, and the patient remained well months after discharge. A review of the published cases revealed 11 cases of *M. hominis* IE, with 72.7% of patients being male, with a mean age of 45 years and a history of valve surgery in all patients. The diagnosis was performed by culture in 54.5% of cases or with the aid of molecular techniques in 45.5%. The prognosis was favorable in 72.7% of patients. The most commonly used antimicrobials were doxycycline, quinolones, and clindamycin.

Blood culture negative IE is commonly caused by *Coxiella burnettii*. In their study, Papakonstantinou et al. present a case of cardiac-implanted electronic device (CIED)-related blood-culture-negative IE that was caused by *C. burnetii* [12]. More specifically, a 54-year-old male with a previous implantation of an ICD three years before was admitted to the hospital due to low-grade fever, prolonged fatigue, and weight loss. The echocardiography showed a large echogenic mass on the ventricular pacing wire in the right ventricle. Repeated blood cultures were negative. Transvenous lead extraction was performed, while a surgical replacement of the tricuspid valve was carried out due to multiple vegetations on the tricuspid valve. Serology showed increased phase I and phase II IgG antibodies, leading to a definite diagnosis of CIED infection. 

*Pasteurella* spp. are facultative anaerobic, Gram-negative coccobacilli that are commonly found in the oral cavity and the gastrointestinal tract of some animals. They are known to cause infections such as septicemia or, more commonly, skin and soft tissue infections in the context of an animal bite [13]. In their systematic review, Alifragki et al. aimed to systematically review all cases of IE caused by *Pasteurella* spp. in the literature [14]. A systematic search of PubMed, Scopus and the Cochrane Library through December 2021 led to the recovery of 28 studies, providing data on epidemiology and clinical and microbiological characteristics, as well as data from 28 patients on the treatment and outcomes of IE by *Pasteurella* spp. A prosthetic valve was present in 21.4% of patients. The most commonly involved intracardiac site was the aortic valve, while the most common clinical presentations were fever, sepsis, septic shock, and heart failure. The most commonly used antimicrobials were cephalosporins, aminopenicillins, and penicillin, and the overall mortality was 17.9%.

This Special Issue includes many interesting papers that will increase the reader’s understanding of IE. As a Guest Editor, I would like to thank all the authors for their contributions, the reviewers for providing high-quality feedback, and the *JCM* editorial team for their support and assistance with this Special Issue.
